# Optimal sensitometric curves of Kodak EDR2 film for dynamic intensity modulated radiation therapy verification

**DOI:** 10.2349/biij.4.1.e4

**Published:** 2008-01-01

**Authors:** S Suriyapee, N Pitaxtarnin, S Oonsiri, C Jumpangern, I Israngkul Na Ayuthaya

**Affiliations:** Division of Radiation Oncology, Department of Radiology, Faculty of Medicine, King Chulalongkorn Memorial Hospital, Bangkok, Thailand

**Keywords:** IMRT treatment plan verification, EDR2 film, sensitometric curves

## Abstract

**Purpose:**

To investigate the optimal sensitometric curves of extended dose range (EDR2) radiographic film in terms of depth, field size, dose range and processing conditions for dynamic intensity modulated radiation therapy (IMRT) dosimetry verification with 6 MV X-ray beams.

**Materials and methods:**

A Varian Clinac 23 EX linear accelerator with 6 MV X-ray beam was used to study the response of Kodak EDR2 film. Measurements were performed at depths of 5, 10 and 15 cm in MedTec virtual water phantom and with field sizes of 2x2, 3x3, 10x10 and 15x15 cm^2^. Doses ranging from 20 to 450 cGy were used. The film was developed with the Kodak RP X-OMAT Model M6B automatic film processor. Film response was measured with the Vidar model VXR-16 scanner. Sensitometric curves were applied to the dose profiles measured with film at 5 cm in the virtual water phantom with field sizes of 2x2 and 10x10 cm^2^ and compared with ion chamber data. Scanditronix/Wellhofer OmniPro^TM^ IMRT software was used for the evaluation of the IMRT plan calculated by Eclipse treatment planning.

**Results:**

Investigation of the reproducibility and accuracy of the film responses, which depend mainly on the film processor, was carried out by irradiating one film nine times with doses of 20 to 450 cGy. A maximum standard deviation of 4.9% was found which decreased to 1.9% for doses between 20 and 200 cGy. The sensitometric curves for various field sizes at fixed depth showed a maximum difference of 4.2% between 2x2 and 15x15 cm^2^ at 5 cm depth with a dose of 450 cGy. The shallow depth tended to show a greater effect of field size responses than the deeper depths. The sensitometric curves for various depths at fixed field size showed slightly different film responses; the difference due to depth was within 1.8% for all field sizes studied. Both field size and depth effect were reduced when the doses were lower than 450 cGy. The difference was within 2.5% in the dose range from 20 to 300 cGy for all field sizes and depths studied. Dose profiles measured with EDR2 film were consistent with those measured with an ion chamber. The optimal sensitometric curve was acquired by irradiating film at a depth of 5 cm with doses ranging from 20 to 450 cGy with a 3×3 cm^2^ multileaf collimator. The optimal sensitometric curve allowed accurate determination of the absolute dose distribution. In almost 200 cases of dynamic IMRT plan verification with EDR2 film, the difference between measured and calculated dose was generally less than 3% and with 3 mm distance to agreement when using gamma value verification.

**Conclusion:**

EDR2 film can be used for accurate verification of composite isodose distributions of dynamic IMRT when the optimal sensitometric curve has been established.

## INTRODUCTION

The dosimetric verification of intensity modulated radiation therapy (IMRT) requires an accurate delivery of the radiation dose. Radiographic film is a popular dosimeter for determining the two-dimensional dose distribution. It has a good spatial resolution which is due to the small grain size and the small aperture of the light beam of the densitometer. However, the film response for photon beams is not considered to be an accurate dosimeter due to the variation of the film response with the energy, depth and field size. In the IMRT beam, the fluence spectrum varies significantly across a single fluence map from the combined in-field and outer-penumbral areas of beamlets. Also the field sizes vary from field to field and from patient to patient. The increase in the low energy photon spectrum in the penumbral region as depth increases can cause a significant increase in film response due to the photoelectric effect of silver bromide in the photographic emulsion [1]. To use film in dosimetry verification of the IMRT plan, Kodak Extended Dose Range 2 (EDR2) film was introduced. It is a very low speed, fine grained film. The silver content of EDR2 film is about one-half that of Kodak XV2 film so the sensitivity of the film is lower. The EDR2 film can be exposed to a dose of at least 300 cGy [2]. The decrease in the emulsion thickness of EDR2 films should decrease the dependence of the film response with depth, field size and energy. Zhu *et al*. [3] and Esthappan *et al*. [4] showed the lower response of EDR2 film with depth, field size and energy compared to XV film. However, for smaller fields of less than 4×4 cm^2^ which are important in IMRT, few data are available for the response of EDR2 film. The other problem associated with film dosimetry is the significant effect of processing conditions, namely type of processor, processing time and temperature [5]. The scanner type also has an effect when reading the optical density. The solution to using film as a good verification tool is to reduce the error associated with the dependence on depth, field size and environmental conditions of the processor and scanner.

Even though other two-dimensional tools have been introduced for IMRT verification such as Gafchromic External Beam Therapy (EBT) film, two-dimensional (2D) diode array or ionisation array, limitations are still observed. The variation in the spatial uniformity of the EBT film limits the accuracy and precision of the results [5]. The disadvantage of the 2D diode array is the spatial resolution, even though new software with a stepper platform can deliver multiple beams with the diode array in different positions providing more measurement points that can be superimposed to give a higher resolution measurement array [6]. However, film is superior in terms of higher resolution which is advantageous for IMRT dosimetry verification.

In this study, the sensitometric curves of EDR2 film were investigated for field sizes of 2×2, 3×3, 10×10 and 15×15 cm^2^ at depths of 5, 10 and 15 cm to select the optimal depth and field size for dosimetric film calibration for dynamic IMRT verification. The idea was to irradiate a single calibration film with one depth and one field size for the range of doses used in the IMRT plan. The error due to the processing conditions and the quality of the densitometry system would be examined and eliminated.

## MATERIALS AND METHODS

### Preparation of densitometer and film processor

The Vidar VXR-16 automatic film scanning densitometer with Scanditronix/Wellhofer OmniPro^TM^ IMRT software was calibrated with a Kodak step wedge film to define the relationship between the densitometer signal and the net optical density. The film scanner operates with a resolution of 142 dots per inch (0.179 mm/pixel) and a depth of 16 bits. The special step wedge film was delivered from the manufacturer with an optical density range from 0.04 to 3.65. The reference density value for each step of the step wedge film was entered into the automatic film scanning densitometer and the graph of the signal versus the net optical density was plotted.

To reduce the effect of film processor conditions, daily quality control of the Kodak RP X-OMAT Model M6B automatic film processor has been performed with a Kodak process control sensitometer. This sensitometer consists of a stable light source, timer, diffusion panel, optical step wedge and pressure plate to eliminate the air gap between the film contacts during exposure. When a film is exposed on both sides to light from the device, the optical step wedge provides a series of light intensities. The film is processed and curves are plotted of step number versus optical density. For routine processor monitoring, three measurements of optical density should be made [7]. One measurement is the base plus fog density and should be made in a region of the film that is not exposed to the light. The second measurement is obtained in a region of the wedge image where the optical density is near 1.0 over the base plus fog density. This is referred to as the speed index. The third measurement should be made in a region of the wedge image where the density is about 0.25 and 2.0 over the base plus fog density. The density difference of these steps is denoted as the contrast index. Moreover, the temperature of the developer solution should be noted. To establish the average baseline values for these variations, measurements were taken for five films. The tolerance values were set so that the measurements of these parameters for the quality assurance (QA) film were compared with the reference and tolerance values of: Speed Index 1.18 ± 0.2, Contrast Index 1.85 ± 0.2, base+fog 0.18 ± 0.03, and Temperature Index 34.8 ± 0.3 to confirm the consistency of the processor performance. When the daily QA film measurement had been performed with the parameter values within the tolerance limits, then the dosimetry film could be developed.

### Reproducibility of results with film

The effect of film processor and the reproducibility of results with film were investigated by irradiating each film at the depth of maximum dose (dmax), in MedTec virtual water phantom using 3x3 cm^2^ field size with the dose ranging between 20 and 450 cGy at 100 cm source-to-axis distance (SAD). This study has been performed nine times. The sensitometric curves were plotted on the same graph to illustrate the deviation of the responses of the films developed at the different times.

### Irradiation of film and sensitometric curves

The dose-response curves of the Kodak EDR2 film to 6 MV X-ray beams from a Varian Clinac 23EX linear accelerator were studied for depths of 5, 10 and 15 cm and field sizes of 2×2 cm^2^, 3×3 cm^2^, 10×10 cm^2^ and 15×15 cm^2^ with delivered doses of 20-450 cGy. Small and moderate field sizes were chosen to investigate the film responses. The maximum dose of the composite IMRT plan could be raised to 400 cGy, so doses of 20-450 cGy were selected for the study. The 25.4×30.5 cm^2^ EDR2 films were irradiated in virtual water phantom; the films were placed at 100 cm SAD, and sandwiched between virtual water phantom slabs. Each phantom slab had dimensions of 30×30 cm^2^ with various thicknesses. The backscatter layers were kept at 20 cm. The EDR2 films were oriented normal to the central axis of the beam.

The optical densities along the central axis were measured using the film scanner. The net optical densities were obtained by subtracting the optical density corresponding to base+fog. Sensitometric curves were plotted as a function of net optical density versus dose for the set of fixed depths and various field sizes and the set of fixed field sizes and various depths for each energy studied.

### Measurement of beam profile

Beam profiles were measured with films in MedTec virtual water phantom for field sizes of 2x2 and 10x10 cm^2^. This phantom agrees with water within 0.5% for 6 MV X-ray beams. The films were placed between slabs of virtual water phantom at 5 cm depth. The central axis of the beam was perpendicular to the surface of the phantom. The beam profiles were plotted by applying the sensitometric curve to convert the optical density to dose and by normalising doses at the off-axis points to the dose at the central axis. Then the beam profiles at 5 cm depth measured with the Scanditronix/Wellhofer CC13 0.13 cc ion chamber in the Scanditronix/Wellhofer 3D water phantom system were compared with beam profiles measured with film at the same field size.

### Optimal calibration curve and verification of clinical IMRT plan for the dependence

After analysing the data, the optimal field size and depth were obtained and used to construct a calibration curve to convert the optical density to dose. The aim was to irradiate a single film with many doses at one definite field size and depth for each set of IMRT plan verifications. To acquire the correct doses of the small field having a few centimetres of space between each other in the same film, the individual doses delivered to small regions at the optimal depth were measured with the Scanditronix/Wellhofer model CC13 0.13 cc ionisation chamber and Scanditronix/Wellhofer model DOSE1 electrometer in virtual water phantom. Then the monitor unit for the delivered dose was calculated and used to irradiate the calibration film. The treatment plan for each IMRT patient was transferred to the virtual water phantom and exposed as the composite field in the coronal plane, at optimal depth close to the isocentre of the patient. All fields were at the same zero gantry angle. This treatment plan used the same beam fluences, energies and monitor units as in the patient plan. The phantom with EDR2 film was subsequently irradiated. Then the absolute isodose distribution in each IMRT plan was compared with the calculated isodose distribution from Eclipse treatment planning using Scanditronix/Wellhofer OmniPro^TM^ IMRT software.

## RESULT

### Calibration of densitometer

**Figure 1:**
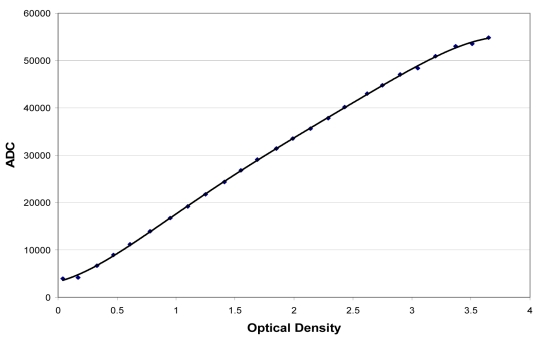
Densitometer calibration curve.

The relationship between the signal of the analogue-to-digital converter (ADC) and the optical density from the step wedge is shown in Figure 1. This relationship was almost linear for optical densities between 0.2 and 3.0. Saturation of the signal began at an optical density of about 3.0. This result indicates that the optical density of the film used should not be more than 3.0.

### Reproducibility of results with film

**Figure 2:**
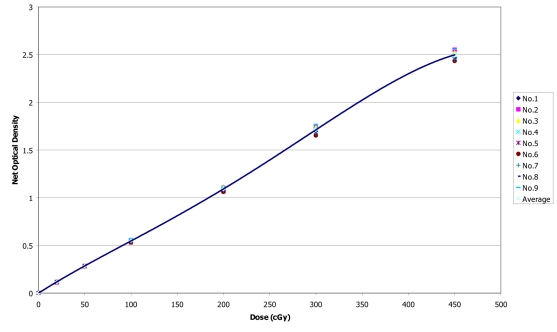
Sensitometric curve for nine measurements with a 3×3 cm^2^ field, dmax at 100 cm SAD.

The reproducibility of results with film is shown in (Table 1) for nine measurements with doses ranging from 20 to 450 cGy and the sensitometric curves are shown in Figure 2. The fitted line represents the average for all data. The percent standard deviations increased with higher doses to a maximum of 4.9%. Good reproducibility and accuracy of results were obtained with film when the irradiated doses were between 20 and 200 cGy; the reproducibility was within 1.9%.

**Table 1:** The average optical density and % standard deviation for 6 measurements of 3×3 cm^2^ field, 1.5 cm d_max_ for 6 MV and 10 MV x-ray beams.

**DosecGy**	**6 MV**		**10 MV**	
	**AverageOD**	**%SD**	**AverageOD**	**%SD**
20	0.209	0.3	0.209	0.5
50	0.349	0.7	0.341	0.8
100	0.588	1.2	0.583	1.0
200	1.130	2.2	1.054	1.3
300	1.752	3.9	1.739	2.9
450	2.537	5.0	2.556	4.2

### Response of film with fixed depths and varying field sizes

**Figure 3:**
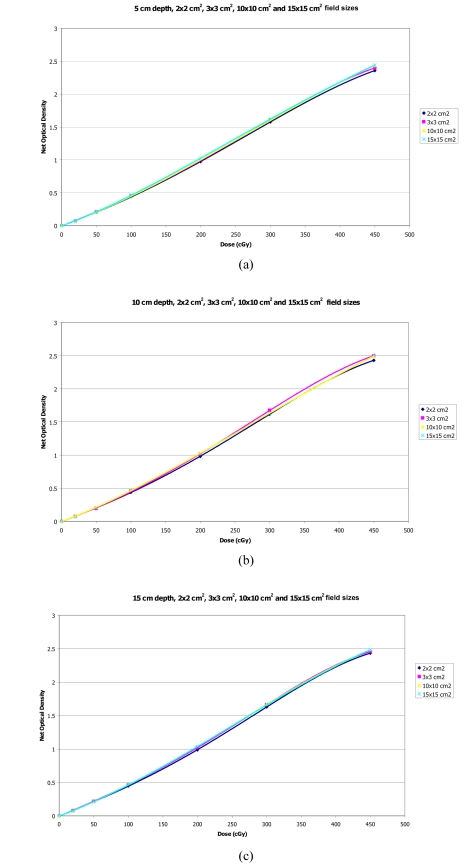
Sensitometric curves for varying field sizes of 2×2 cm^2^, 3×3 cm^2^, 10×10 cm^2^ and 15×15 cm^2^ with fixed depths of (a) 5 cm, (b) 10 cm and (c) 15 cm.

Sensitometric curves were plotted as a function of net optical density versus dose for field sizes of 2×2 cm^2^, 3×3 cm^2^, 10×10 cm^2^ and 15×15 cm^2^ with fixed depths of 5, 10 and 15 cm as shown in Figures 3a, 3b and 3c, respectively. The marker points in the figure represent measured data points and the solid lines illustrate the fitted data.

All of the sensitometric curves for fixed depth and with various field sizes showed the difference in the film response with field size when the dose was increased. The effect of field size tended to be greater at shallow depths (5 cm) than at deeper depths (15 cm). The maximum film response differences between 2x2 and 15×15 cm^2^ fields at a dose of 450 cGy were within 4.2%, for the fixed depths studied.

### Response of film with fixed field sizes and varying depths

**Figure 4:**
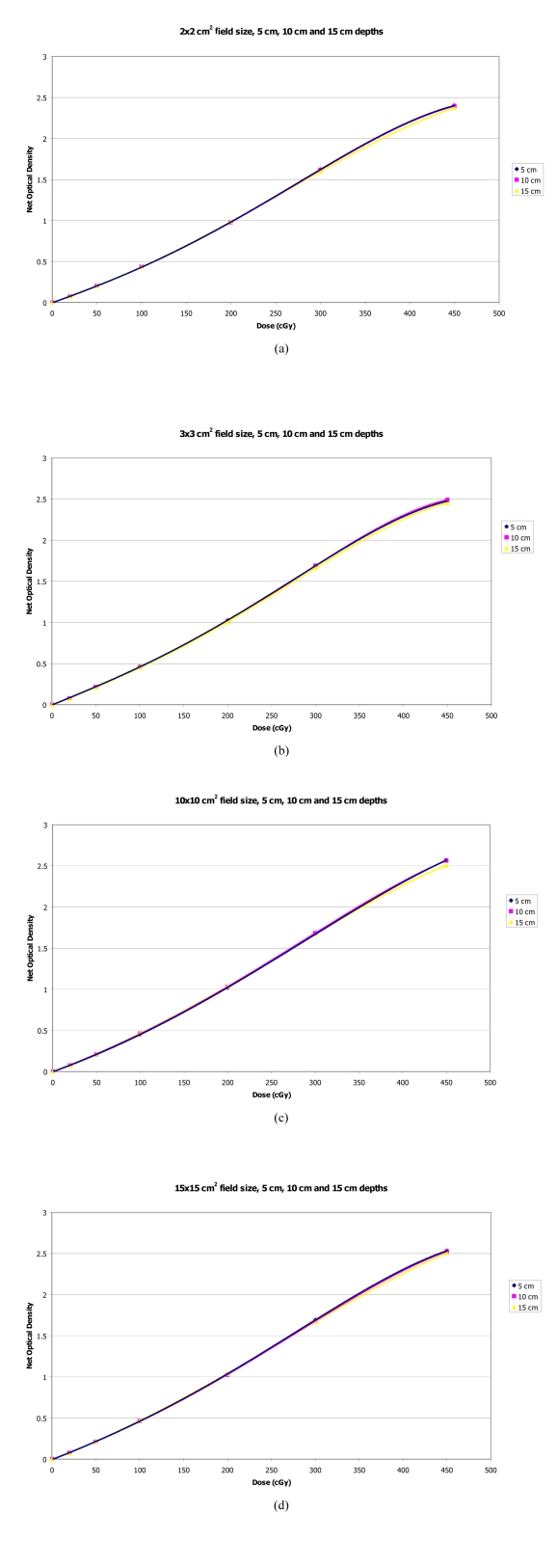
Sensitometric curves for varying depths of 5, 10 and 15 cm with fixed field sizes of (a) 2×2 cm^2^, (b) 3×3 cm^2^, (c) 10×10 cm^2^ and (d) 15×15 cm^2^.

Sensitometric curves for depths of 5, 10 and 15 cm with fixed field sizes of 2×2 cm^2^, 3×3 cm^2^, 10×10 cm^2^ and 15×15 cm^2^ are shown in Figures 4a, 4b, 4c and 4d respectively. The curves showed less difference of film response between the different depths for all fixed field sizes studied compared with the effect of different field sizes for fixed depths. The maximum film response differences between 5 and 15 cm depth at a dose of 450 cGy were within 1.8%, for the fixed field sizes studied.

### Beam profile

**Figure 5:**
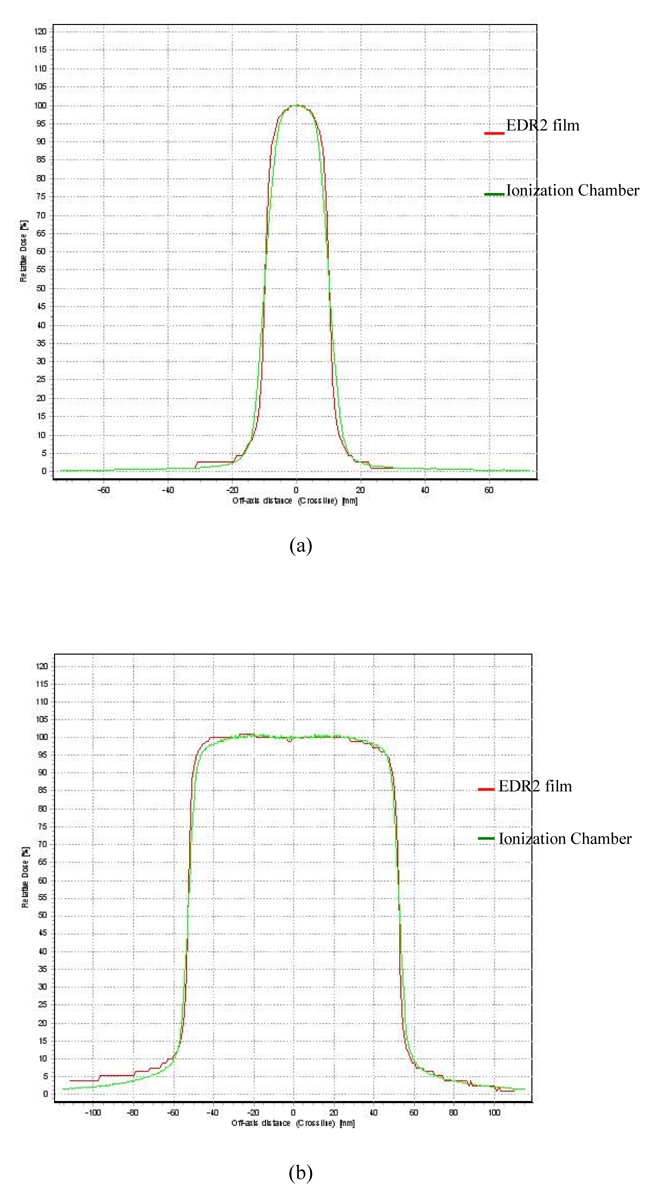
Dose profile measured with EDR2 film in solid water phantom and an ion chamber in water phantom for (a) 2×2 cm^2^ field and (b) 10×10 cm^2^ field at 5 cm depth.

Comparisons of beam profiles between film and ionisation chamber measurements for field sizes of 2×2 and 10×10 cm^2^ at 5 cm depth are shown in Figures 5a and 5b, respectively. The doses for the film were obtained from the sensitometric curves and these were then normalised to the dose at the central axis. The dose beam profiles measured with the film were superimposed on the dose beam profile measured with the ionisation chamber in the water phantom. The agreement of profiles for small and large field sizes was within 2 mm. These results agreed with other studies [3,4].

### Optimal film calibration curve for IMRT dosimetry verification

The goal of film calibration is to convert a film density value obtained from an actual beam measurement into an accurate tissue dose value. As IMRT field size and fluence maps vary from field to field, from treatment site to treatment site, and from patient to patient, there is no simple way of modelling such variability of fluence maps within a phantom and incorporating such a model in a calibration procedure. Therefore, in general, the use of a relatively small field size, such as 6×6 cm^2^ or 7×7 cm^2^, for calibration is recommended [8].

For our study, after the dosimetric properties of EDR2 film had been investigated, a small field size of 3×3 cm^2^ was selected using multileaf collimators to generate regions for doses from 20 to 450 cGy on a single sheet of film. The film was placed in the virtual water phantom at an isocentre of 5 cm depth perpendicular to the beam. These parameters were taken to be the optimal conditions for the film calibration curves for six X-ray beams. The 3×3 cm^2^ field was chosen due to the reliable dose measurement when using the 0.13 cc ion chamber for output measurement. However, 3×3 cm^2^ and 2×2 cm^2^ field sizes gave sensitometric curves which were not significantly different over the range of doses and depths studied. Small fields were chosen because most of the IMRT fields are composed of small fields and a significant difference in film response was found when compared with the larger fields of 10×10 and 15×15 cm^2^. The 5 cm depth was selected because it was close to the isocentre depth of head and neck cancer which is mostly treated in this institute. The beam profile for a small field measured with film at 5 cm depth agreed with that measured with the ionisation chamber.

### Verification of clinical IMRT plan

**Figure 6:**
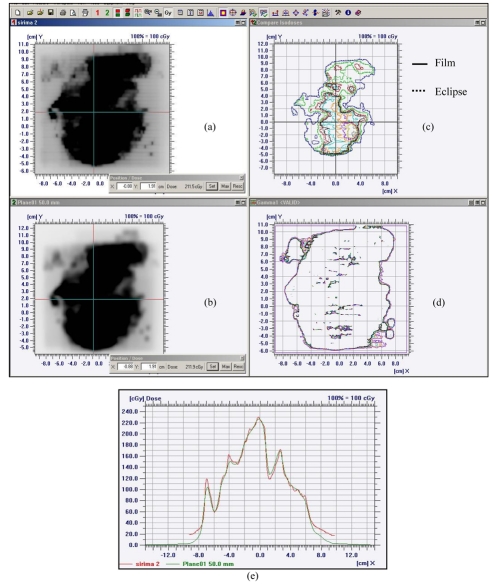
Verification of dose distribution calculated by Eclipse treatment planning and measured by EDR2 film for 6 MV X-ray beams: (a) Fluence map from film measurement; (b) Fluence map from Eclipse calculation; (c) Absolute isodose distribution comparison; (d) Gamma value verification and (e) Profile comparison between EDR2 film and Eclipse treatment planning.

The calibration curve was measured for the dose range of 20 to 450 cGy for every IMRT plan verification. An example of verification of the IMRT plan in the virtual water phantom for composite fields at the same gantry angle of zero degrees was demonstrated using Scanditronix/Wellhofer OmniPro^TM^ IMRT software by looking at the absolute dose distribution, beam profile and gamma evaluation [9] as shown in Figure 6.

The fluence map obtained from film in Figure 6a looks visually similar to the fluence map from Eclipse calculation in Figure 6b. The cross-hair in both fluence maps is the reference point for alignment. The isodose distribution in Figure 6c shows the overlap between the solid line of the EDR2 film measurement and the dotted line of the calculation. The gamma evaluation in Figure 6d is shaded if the gamma values are more than 1 with the criteria of 3% dose difference and 3 mm distance to agreement. The profile in Figure 6e shows a reasonable match between measurement and calculation apart from the penumbral region where the planning system model underestimated the dose. The measured film profile shows better resolution than the calculated profile because the resolution of film was 0.2 mm while the treatment planning was 2 mm.

During the period October 2005-May 2007, about 200 IMRT plans for nasopharynx, lung, prostate and other cancers were verified by EDR2 film. Most of the plans showed good agreement between the measured and calculated dose in the central region but some higher doses were observed from film at the edge of the beams. This result illustrates the greater response of film in the low energy area and also that the algorithm calculated by treatment planning was underestimated. The gamma values were mostly less than 1 except the area near the edge of the field. The five dose points from film at positions (0, 0), (0, 2), (0, -2), (2, 0) and (-2, 0) for 42 IMRT plans were read [10], and the mean difference from calculation of these points was 2.4% with a standard deviation of 1.07. The result demonstrated the accuracy of IMRT dose distribution using EDR2 film which implied good film calibration curves.

## DISCUSSION AND CONCLUSIONS

Film can be used for the determination of relative or absolute dose distributions if a suitable calibration curve is available. In principle, absolute dose values are obtained if the film sensitivity is identical for all films. But in a real situation, the film sensitivity is rarely identical due to the variation in emulsion coating. The same batch of film should be used. The processing conditions affect the film response, and investigation of the processor equipment type, chemicals, processing time and temperature should be considered [5]. There are also variations in film densitometer measurements which lead to inaccuracies in optical density readings. However, film sensitivity is dependent on energy, depth, and field size, and the conversion of dose is not a simple procedure. To determine a suitable calibration curve for converting optical density to absolute dose, these parameters need to be studied so that the optimal calibration curve will give the correct dose value in all patients in the IMRT field.

The EDR2 films used in this study came from the same batch to reduce the deviation of sensitivity due to variation in emulsion coating thickness. A small difference in film optical density response would be obtained for repeated irradiation and for films developed at different times if the complete daily QA had not been performed before developing film dosimetry. For accurate determination of the absolute dose distribution in the IMRT plan verification, the doses given to the film should be in the order of 200 cGy so that we can measure the absolute dose with an accuracy within 2.0%. At the same time, using EDR2 film for the range of depths and field sizes investigated here, our results indicated that for megavoltage photon beams, sensitometric curves are slightly dependent on field size and depth of calibration. The difference in film response due to field size and depth was within 2.5% for doses from 20 to 300 cGy. Chetty and Charland [11] reported comparable results for the variation of optical density in the order of 2-3% for field sizes of 3x3 cm^2^ and 10x10 cm^2^ at dmax values of 5 and 15 cm. This implies that the field size and depth have a slight effect on the response of the film. The reduction in silver content and smaller grain size of EDR2 film reduced the energy dependence problem. The results were confirmed by Yeo and Kim [8] who mentioned that EDR2 film is less sensitive to scattered low energy photons.

A calibration curve was generated in a single sheet to convert film optical density to dose with exposures from 20 to 450 cGy, 3x3 cm^2^ field size, 5 cm depth and 100 cm SAD. The advantage of choosing these parameters as a single film is because it can be irradiated and developed at the same time so there is no effect of film development.

If a composite IMRT plan was selected such that the average daily fraction dose was in the order of 200-300 cGy, the difference between measured and calculated dose was generally less than 3%. On the other hand, if the average dose went up to 300-400 cGy, the difference could increase up to 6% because the effect of field size was dominant at doses higher than 300 cGy and also because of the inaccuracy of the densitometer readings at higher optical densities. However, the dose selected to calibrate the film must be high enough to cover the maximum dose in the IMRT plan. The dose range would be adjusted according to the absolute isodose distribution in the planning because, for each calibrated film set, only eight levels of dose could be irradiated. The response of the film with the actual gantry angle showed agreement with the calculation in the same direction as the beam at zero gantry angle [9]. The point dose measured by the ionisation chamber at the same time as film measurement showed good agreement. For 200 IMRT plans, we were quite successful in IMRT verification with film. Based on our experience, we believe that EDR2 film is the tool of choice for IMRT plan verification.
